# A comparison of the efficiency of 22G *versus* 25G needles in EUS-FNA for solid pancreatic mass assessment: A systematic review and meta-analysis

**DOI:** 10.6061/clinics/2018/e261

**Published:** 2018-01-11

**Authors:** Hugo Gonçalo Guedes, Diogo Turiani Hourneaux de Moura, Ralph Braga Duarte, Martin Andres Coronel Cordero, Marcos Eduardo Lera dos Santos, Spencer Cheng, Sergio Eiji Matuguma, Dalton Marques Chaves, Wanderley Marques Bernardo, Eduardo Guimarães Hourneaux de Moura

**Affiliations:** Divisao de Endoscopia Gastrointestinal, Faculdade de Medicina FMUSP, Universidade de Sao Paulo, Sao Paulo, SP, BR

**Keywords:** Pancreatic Cancer, Endoscopic Ultrasonography, Fine Needle Aspiration Biopsies

## Abstract

Our aim in this study was to compare the efficiency of 25G *versus* 22G needles in diagnosing solid pancreatic lesions by EUS-FNA. We performed a systematic review and meta-analysis. Studies were identified in five databases using an extensive search strategy. Only randomized trials comparing 22G and 25G needles were included. The results were analyzed by fixed and random effects. A total of 504 studies were found in the search, among which 4 randomized studies were selected for inclusion in the analysis. A total of 462 patients were evaluated (233: 25G needle/229: 22G needle). The diagnostic sensitivity was 93% for the 25G needle and 91% for the 22G needle. The specificity of the 25G needle was 87%, and that of the 22G needle was 83%. The positive likelihood ratio was 4.57 for the 25G needle and 4.26 for the 22G needle. The area under the sROC curve for the 25G needle was 0.9705, and it was 0.9795 for the 22G needle, with no statistically significant difference between them (*p*=0.497). Based on randomized studies, this meta-analysis did not demonstrate a significant difference between the 22G and 25G needles used during EUS-FNA in the diagnosis of solid pancreatic lesions.

## INTRODUCTION

The most common etiology of solid pancreatic masses is adenocarcinoma, responsible for 85 to 95% of cases 1. Computed tomography (CT) and magnetic resonance imaging (MRI) are the best options for the initial evaluation of these lesions, as they have the ability to detect the presence of distant metastases and affected adjacent lymph nodes. However, in a prospective randomized trial comparing endoscopic ultrasound (EUS) and CT for the diagnosis of pancreatic cancer, DeWitt et al. confirmed that CT is less sensitive in the diagnosis of pancreatic cancer and is equivalent to EUS when evaluating lymph node staging of the disease 2.

EUS is currently considered the most reliable and accurate method for the detection and diagnosis of pancreatic masses. The reported sensitivity of EUS in the detection of pancreatic cancer is between 94% and 100%. Compared with CT, EUS can detect up to 14% of pancreatic tumors that are not visualized by CT, especially tumors that are smaller than 20 mm, which are associated with higher rates of diagnostic failure by MRI and CT. Thus, EUS has greater test sensitivity and accuracy 3.

EUS-guided fine-needle aspiration has progressed in relation to its use in studying pancreatic masses based on the ability to safely collect material for cytological and/or anatomopathological analysis with a low complication risk of 0.5 to 2%. The main risks are bleeding and acute pancreatitis 4.

To standardize the EUS fine-needle aspiration (EUS-FNA) procedure, Bang et al. have proposed the following algorithm for needle selection: 25 Gauge (G) needles for transduodenal EUS-FNA, 22G or 25G needles for other punctures, 19G flexible needles for transduodenal interventions and standard 19G needles for other access routes 5.

Currently, EUS-FNA needles are available as 19G, 22G and 25G needles 6, with an estimated diagnostic sensitivity of 85-93% 7. The 25G needles are malleable, not interfering with the torque of the device, but they are less likely to aspirate a suitable quantity of material for anatomopathological analysis. In general, studies have reported good results using the 22G needle 8, but there is no evidence to date of its high scientific value to prove its superiority over the 25G needle 9.

The decision to use a specific needle gauge involves the associated risks and benefits. The greater the caliber of the needle, the greater the risk of post-puncture bleeding and the greater the chance of obtaining aspirated blood, which may compromise the quality of the sample, reducing its diagnostic value 10.

A smaller needle is technically easier to handle within the device, especially in anatomical areas where the position of the endoscope has not been optimized, such as in the duodenum where the head of the pancreas is examined.

The 25G needle, because of its flexibility, may present benefits compared with larger needles used for EUS-FNA at more difficult access sites, such as the pancreatic head, uncinate process and proximal region of the bile duct 11. For example, one study reported a failure to puncture in 33% of cases with lesions located at the uncinate process 12. Thus, most studies compare only 22G and 25G needles, but there is still no clear evidence of the superiority of one needle over the other.

We have identified several studies on EUS-guided fine-needle aspiration and methods for its improvement. These studies have answered several questions, but there is no strong evidence to date regarding which needle is best.

The objective of this study was to compare the success rates of 22G and 25G needles in diagnosing the malignancy of solid pancreatic lesions when performing EUS-FNA.

## MATERIALS AND METHODS

### Protocol and registration

A protocol specifying the eligibility criteria and methods of analysis for the studies included in this systematic review and meta-analysis was established and documented prior to the start of this review, and it can be accessed at http://www.crd.york.ac.uk/PROSPERO with the registration number CRD42016046810.

### Eligibility criteria

Types of studies: Randomized trials comparing the use of 22G and 25G needles for EUS-FNA of solid pancreatic lesions were included.

Types of participants: Patients with solid pancreatic lesions.

Types of interventions: EUS-FNA with 22G and 25G needles. The gold standard for the comparison of the two methods was the result of the anatomopathological analysis of the biopsies or resected parts of the suspicious lesions identified by EUS-FNA or the clinical diagnosis after a follow-up of at least 6 months.

Types of outcome measures: The efficiency indices of the methods were evaluated, such as sensitivity, specificity, positive predictive value (PPV), negative predictive value (NPV) and accuracy.

### Information sources

Searches to select articles were carried out in electronic databases and in reference articles related to the 22G and 25G needle in EUS-FNA for solid pancreatic mass. The databases used were the following: Medline, Scopus, Cochrane Central Register of Randomized Controlled Trials/CENTRAL, Latin American and Caribbean Health Sciences (LILACS) and Cumulative Index to Nursing and Allied Health Literature (CINAHL). The date of the last search in all the databases was 03/31/2017.

### Search

The search strategies used varied by database and are specified below:

- Medline: (((((Biops* OR Cytological Techniques OR cytolog* OR Gauge OR needle OR 22 OR 25))) AND (((endosonography OR endoscopic ultrasonography OR endoscopic ultrasound OR EUS OR Endoscopic Ultrasound-Guided Fine Needle Aspiration))))) AND random*.

- LILACS, Scopus, CINAHL, Cochrane/CENTRAL: (endosonography OR endoscopic ultrasonography OR endoscopic ultrasound) AND pancreas AND (22G OR 25G).

### Study selection

The articles were initially selected by evaluation of the titles and abstracts, followed by evaluation of the pertinence of the complete text. This process was performed by two independent, non-blinded reviewers. Differences were resolved through discussion and consensus with the participation of all authors.

Non-randomized studies and abstracts were excluded from the meta-analysis.

### Data collection process

Data were collected from absolute numbers provided directly or from inferred information reported throughout the text. The data were placed in 2 x 2 tables, where the true positives, false positives, true negatives and false negatives were separated. Each data collection process was performed by two independent authors and reviewed by all authors. The differences were resolved through discussion and consensus among the authors. When the data were not clear in the paper, we attempted to contact the authors by personal and institutional email.

### Data items

The criteria considered for the positivity of the methods in the meta-analysis were the same as those established by the authors. We did not consider the number of punctures performed, the technique used (vacuum or not), or the presence or not of the cytopathologist in the room (ROSE) in the analysis.

### Risk of bias in individual studies

The risk of bias was assessed through the Quality Assessment of Diagnostic Accuracy Studies (QUADAS-2) and the JADAD scale.

All non-randomized studies were excluded and were considered inadequate in the evaluation of the risk of biases of the main test when the endoscopist knew the results of the pathology prior to the EUS-FNA puncture. Regarding the applicability of the methods, we evaluated whether there was standardization of well-established and adequate criteria to consider a puncture positive. Regarding the risk of bias and the applicability of the gold standard examination, the pathologist should be blinded to the type of needle used in the positive findings.

To assess the risk of flow bias, we considered appropriate prospective studies, the patients in which should be randomized to one of the groups with examinations performed at the same time and with similar imaging technology. We also evaluated whether the gold standard method, the anatomopathological analysis, was performed in all patients and whether all the lesions were identified.

### Summary measures and planned methods of analysis

In the meta-analysis, we calculated the sensitivity, specificity, and negative and positive likelihood values for each study using the Mantel-Haenszel fixed-effects method 13. Forest plots were then generated in addition to the summary receiver operating characteristic (sROC) curve, and calculations of the areas under the curves were performed. All the variables were assessed in one analysis per patient. A value of 0.1 was added to each cell in the 2x2 tables in place of possible zero frequencies. All statistical tests were binary, and a significance level of 5% was established. To assess heterogeneity, the I-square test was used, where a value greater than 50% was considered as marked heterogeneity. The sROC curves were analyzed using the linear model developed by Littenberg and Moses.

The software used was Meta-DiSc (version 1.4; Unit of Clinical Biostatics, the Ramón y Cajal Hospital, Madrid, Spain) and Review Manager (RevMan) 5.3 software (Copenhagen: The Nordic Cochrane Center, The Cochrane Collaboration, 2011).

## RESULTS

### Study selection

**Figure 1:**
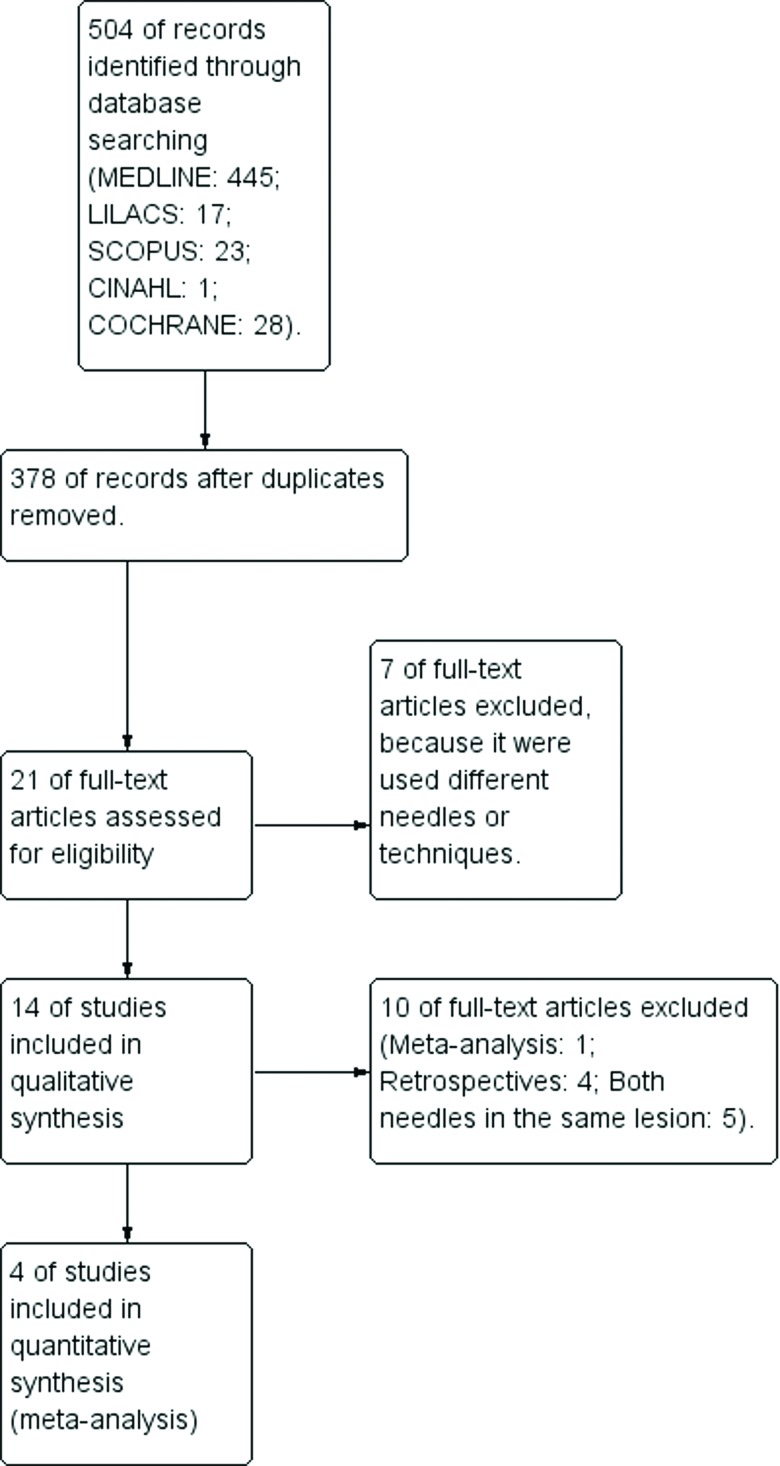
Flow chart of the studies identified with the numbers of studies that were excluded and included in the eventual meta-analysis.

In the literature searches performed through the electronic databases, 504 articles were identified (Figure 1). Of these articles, 483 were excluded after evaluation of the title and the abstract because they had no relation to the theme established for this review or were duplicates. The remaining 21 articles were evaluated in their entirety.

After reading the articles, 7 studies were excluded because they used different needles or techniques 14-20, such as 19G needles, EUS fine-needle biopsy (EUS-FNB), or new needle models. Thus, 14 articles were selected for the systematic review.

Of these 14 articles that were included in the meta-analysis, one was excluded because it was already a meta-analysis 7, four were excluded because they were retrospective studies 21-24, and 5 were excluded because they used both needles in the same lesion 25-29. In the latter case, a second EUS-FNA at the same time for the same lesion could result in more blood contamination than the first puncture, which could be a significant source of bias. All 4 remaining studies consisted of randomized controlled trials and were included in the meta-analysis 9,30-32.

### Study characteristics

The four studies included in this meta-analysis provided all the necessary data to compare the efficiency of the EUS-FNA punctures. A total of 462 patients were evaluated, 233 of whom were punctured with 25G needles and 229 of whom were punctured with 22G needles. All these studies were prospective, randomized and controlled, and the patients were separated into two homogeneous groups, with each group evaluated by fine-needle aspiration puncture with either 22G or 25G needles. The included patients were known to have only pancreatic masses, with varying locations within the organ.

All four studies included in the meta-analysis included patients with a previous diagnosis of solid pancreatic masses, allocated by randomization in different arms for EUS-FNA with 22G or 25G needles.

As the gold standard method, all studies used the lesion anatomopathological evaluation or, when this was not possible, follow-up between 6 30 and 12 months 9.

The criteria for the definition of a solid pancreatic mass varied among the studies. Two studies considered only the findings of images suggestive of a solid pancreatic lesion 9,32. The third study considered the suspected clinical picture, associated with some direct or indirect imaging, and an EUS demonstrating solid lesion content of at least 60% 31. The fourth study was more generic, and any solid lesion adjacent to the gastrointestinal tract and found by any imaging method was punctured, including solid pancreatic lesions. From this fourth study, only those data referring exclusively to solid pancreatic lesions were extracted 30.

Regarding the detailed methods of the EUS-FNA performed to obtain material for anatomopathological analysis, the studies had different techniques with respect to the use of a vacuum, number of punctures performed, number of oscillations performed in each puncture and the presence of a pathologist. These methods are detailed as follows (for more details of the studies, see Table 1):

- Vilmann P et al. 2013: used the equivalent vacuum aspiration of 10 ml (as did the other studies) and performed six “forward-and-backward” movements with the needle at each puncture, but without the presence of a cytopathologist in the room 30.

- Lee JK et al. 2013: used the equivalent vacuum aspiration of 10 ml, and the stylet was present at the time of puncture; however, a cytologist was not present in the room 9.

- Camellini L et al. 2011: kept the stylus for puncture, used a vacuum equivalent to 10 ml, and ensured no more than 10 movements in each puncture. However, upon suspicion of a hypervascularized lesion, the vacuum was not applied. An anatomopathologist was present in the room to evaluate the quality of the material obtained in each puncture and was blinded to the needle gauge used 32.

- Siddiqui UD et al. 2009: used a vacuum by suction with a 10-ml syringe, and a cytologist was present in the room 31.

### Risk of bias within studies

**Figure 2:**
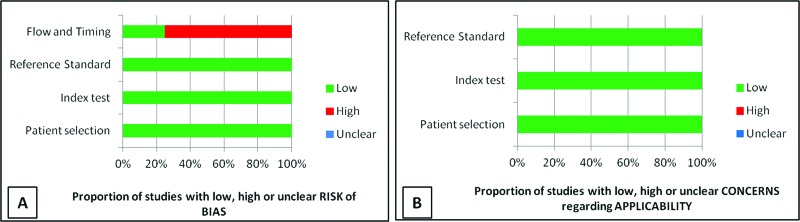
**(A)** Proportion of studies with high, low or inconclusive risk bias, showing only a high risk of bias in the flow and timing; **(B)** Proportion of studies with biases of high, low or non-clear risk applicability. There is a low risk of applicability biases.

All the studies had a low risk of bias with respect to patient selection, the primary test used and the gold standard method used. Moreover, in terms of patient flow and time, the risk was high in 75% of the samples, as three of the four studies had patient losses during follow-up (Figure 2A).

Regarding patient eligibility, the index test and the gold standard method used, the risk of bias was considered low (Figure 2B).

When the quality of the clinical trials was assessed on the JADAD scale, they all had high scores and were considered high-quality studies.

### Analysis of results

**Figure 3:**
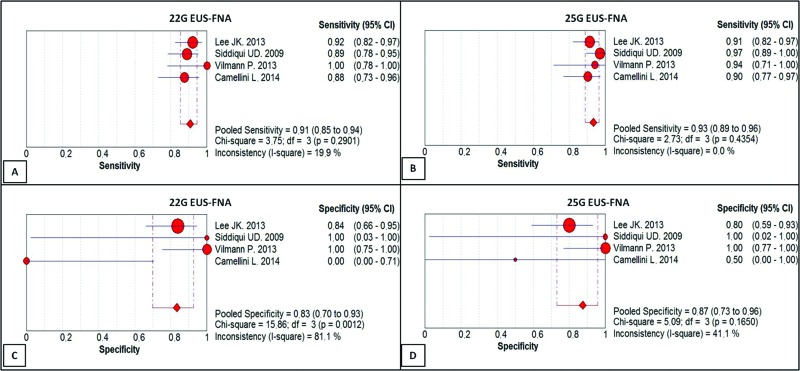
**(A)** Sensitivity of EUS-FNA with the 22G needle (91%); **(B)** Sensitivity of EUS-FNA with the 25G needle (93%); **(C)** Specificity of EUS-FNA with the 22G needle (83%); **(D)** Specificity of EUS-FNA with the 25G needle (87%).

In the per-patient analysis, four studies with a total of 462 patients were analyzed. Among these patients, 344 were diagnosed with malignant tumors of the pancreas, corresponding to 74.45% of the patients. The sensitivity of the 22G needle was 91%, with a confidence interval of 85 to 94% and a heterogeneity of 19.9% (Figure 3A). For the 25G needle, the sensitivity was 93%, with a confidence interval of 89-96% and heterogeneity of 0.0% (Figure 3B). There was no statistically significant difference between the methods.

The specificity of the 22G needle was 83%, with a confidence interval of 70 to 93% and heterogeneity of 81.1% (Figure 3C). The specificity of the 25G needle was 87%, with a confidence interval of 73-96% and heterogeneity of 41.1% (Figure 3D). Again, there was no statistically significant difference between the methods.

**Figure 4:**
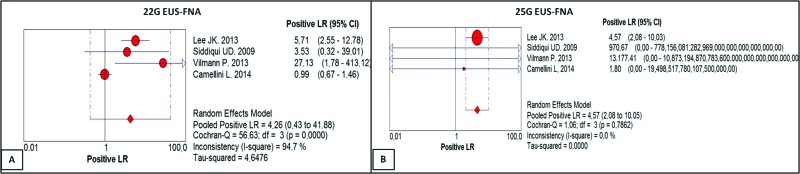
**(A)** Positive likelihood ratio of EUS-FNA 22G. For a given prevalence of 71.61%, the positive likelihood ratio of 4.26 increased the probability 4.26-fold of the result being truly positive rather than false positive, with a post-test probability of 91.3%. **(B)** Positive likelihood ratio of EUS-FNA with the 25G needle. For a given prevalence of 77.25%, the positive likelihood ratio of 4.57 increased the probability 4.5-fold that the result is truly positive rather than false positive, with a post-test probability of 93.85%.

The positive likelihood ratio of the 22G needle score was 4.26, with a confidence interval of 0.43 to 41.88 and a heterogeneity of 94.7% (Figure 4A). For the 25G needle, the positive likelihood ratio was 4.57, with a confidence interval of 2.08 to 10.03 and a heterogeneity of 0.0% (Figure 4B). There was no statistically significant difference between the methods.

**Figure 5:**
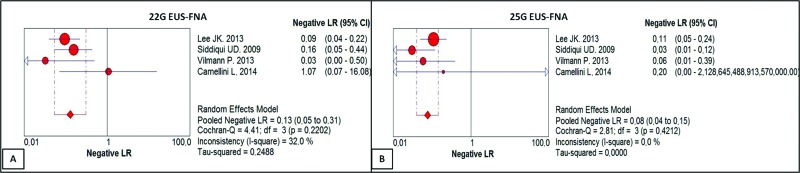
**(A)** Negative likelihood ratio of EUS-FNA with the 22G needle. For a given prevalence of 71.61%, the negative likelihood ratio of 0.13 reduced the probability by 0.13-fold of the result being truly negative rather than false negative, with a post-test probability of -91.3%. **(B)** Negative likelihood ratio of EUS-FNA with the 25G needle. For a given prevalence of 77.25%, the negative likelihood ratio of 0.08 reduced the probability 0.08-fold of the result being truly negative rather than false negative, with a post-test probability of -93.85%.

The negative likelihood ratio of the 22G needle was 0.13, with a confidence interval of 0.05 to 0.31 and heterogeneity of 32.0% (Figure 5A). For the 25G needle, the negative likelihood ratio was 0.08, with a confidence interval of 0.04 to 0.15 and heterogeneity of 0.0% (Figure 5B). There was no statistically significant difference between the methods.

**Figure 6:**
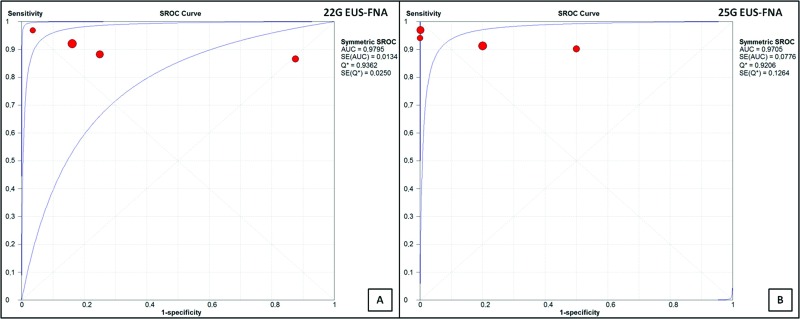
**(A)** sROC curve for EUS-FNA with the 22G needle. Area under the curve of 97.95%. **(B)** sROC curve for EUS-FNA with the 25G needle. Area under the curve of 97.05%.

The post-test probability was 93.85% for the 25G needle and 91.30% for the 22G needle. The area under the sROC curve was 0.9795 for the 22G needle (Figure 6A) and 0.9705 for the 25G needle (Figure 6B). There was no statistically significant difference between them (*p*=0.497).

## DISCUSSION

### Summary of evidence

In our study, the sensitivity of the 25G needle was 93%, while that for the 22G needle was 91%, and there was no statistically significant difference between the needles.

Regarding the statistical interpretation, the positive likelihood ratio (+LR) of EUS-FNA with the 25G needle in our study shows that, for a given prevalence of 77.25%, the +LR of 4.57 increased the probability 4.5-fold that the result is truly positive instead of being a false positive, with a post-test probability of 93.85%. Similarly, for the 22G needle, for a prevalence of 71.61%, the +LR of 4.26 increased this probability 4.26-fold, with a post-test probability of 91.3%.

Meanwhile, the negative likelihood ratio (-LR) of EUS-FNA performed with a 25G needle indicates that, for a given prevalence of 77.25%, the -LR of 0.08 reduces the probability of the result being truly negative rather than being a false negative by 0.08-fold, with a post-test probability of -93.85%. Similarly, for the 22G needle and with a prevalence of 71.61%, the -LR of 0.13 reduces that probability 0.13-fold, with a post-test probability of -91.3%.

Moreover, a positive likelihood ratio greater than 10 provides strong evidence to confirm a positive diagnosis, whereas a negative likelihood ratio of less than 0.1 virtually precludes a negative diagnosis 33. Thus, the negative likelihood ratio in this systematic review and meta-analysis of 0.08 demonstrates that a negative result for a 25G needle is indeed reliable.

Meanwhile, the areas under the sROC curve in the per-patient analysis were 0.9795 for the 22G needle and 0.9705 for the 25G needle, demonstrating good accuracy of the methods, with no statistically significant difference between them.

The best scientific evidence currently available is from a large meta-analysis based on 33 studies 8 that showed that the EUS-FNA method obtained a sensitivity of 85% to 91%, specificity of 94% to 98%, and positive predictive value of 98% to 99%, independent of the needle used for the diagnosis of pancreatic lesions. These results are in accordance with those derived from the present study, and they support the recommendation to use EUS-FNA in the case of pancreatic lesions, as EUS-FNA provides a high pre-test probability for malignancy and negative histopathological diagnosis 11,34,35.

Madhoun et al. 7 published the most recent meta-analysis on this subject in 2013, and of the eight meta-analysis, three were retrospective, five were prospective and only three were randomized and double blind. Nevertheless, the authors found a sensitivity and specificity of 85% and 100% for the 22G needle, respectively. For the 25G needle, the sensitivity values were 93% and 97%, respectively. Moreover, they proved in a linear bivariate analysis that the 25G needle is more sensitive than the 22G needle (*p*=0.0003), although the needles had comparable specificity (*p*=0.97).

The meta-analysis by Madhoun et al. was based on retrospective studies. Despite reporting a heterogeneity of 0.0%, the results were polarized by the study by Yusuf et al., which was also a retrospective study. The allocation of cases to the needles was made based on the availability of the needles. During the initial phase, only 22G needles were available, while 25G needles were used following the initial phase 21. We believe that the cases collected in retrospective studies or the cases in which the lesion was punctured with the two needles directly interfered with this result. For example, the accuracy of one puncture performed after another could be reduced due to the aspiration of a large quantity of red blood cells, especially if the 22G needle was used in the second puncture. In contrast, our meta-analysis involved only randomized controlled trials, with heterogeneities of 0.0% and 19.9% when referring to the sensitivity of the 25G and 22G needles, respectively. In accordance with our results, in 2013, Affoler et al. published a more extensive systematic review that included 19G needles and subgroup analyses 10. This analysis showed no statistically significant difference between needle calibers in the diagnosis of pancreatic lesions, with a sensitivity of 91% for the 25G needle and 78% for the 22G needle. Despite this result, a high heterogeneity for sensitivity was found (I^2^=85.1%), making any comparative analysis between the needles impossible. This is the first meta-analysis comparing such needles that is based only on uniform studies and with a high methodological value. After the statistical analysis, we verified that there was no statistically significant difference in the diagnostic capacity for malignancy of the solid pancreatic masses between the two needles.

As has been noted in several other studies, we found that the larger drilling area of the 22G needle did not translate into samples of higher volume or quality. This conclusion reinforces the idea that efforts to optimize diagnosis by EUS-FNA should perhaps focus on other factors. Moreover, the success of a larger-caliber needle is limited by the anatomy of the target lesion, particularly in pancreatic head and uncinate lesions 29.

The discrepancy in the literature shows that needle selection is a complex process that depends on several factors, such as lesion morphology and location, presence of a cytopathologist in the room, and preferences of the endoscopist 11.

Regarding the possible biases of our study, the QUADAS-2 analysis indicated a high variance in follow-up time and patient flow. For example, not all the patients in the included studies were surgically operated on, and anatomopathological analysis of the surgical specimens as a method of comparison was therefore not performed. Moreover, some of the studies had a follow-up time of six months after diagnosis, which may not necessarily be a sufficient duration to evaluate the long-term disease course.

It should be emphasized that this is a pure meta-analysis, with all the studies involved being randomized, controlled and blind clinical trials of high methodological and statistical value, a type of analysis that has not yet been published on this subject.

This systematic review and meta-analysis did not demonstrate a statistically significant difference between 22G and 25G needles in EUS-FNA for the diagnosis of solid pancreatic lesions.

Therefore, the choice of needle for use in EUS-FNA for solid pancreatic masses must be determined by the physician, who should carefully study the different variables that will affect the tissue sampling, such as material availability, transgastric or transduodenal puncture, size and location of the lesion, and whether the purpose is diagnostic or therapeutic.

## AUTHOR CONTRIBUTIONS

Guedes HG and Bernardo WM performed the systematic review and meta-analysis. Guedes HG, Cordero MA, Duarte RB and Moura DT wrote the article. Moura EG, Santos ME, Cheng S, Matuguma SE and Chaves DM revised the manuscript. The final version of the manuscript was approved by all authors.

## Figures and Tables

**Table 1:** Characteristics of the 4 studies selected for the meta-analysis. All the studies were randomized, prospective and controlled.

Study (year)	Country	Type of study	Patients included 22G/25G	Age, mean 22G/25G	Mean number of passes per lesion per needle	Patients with inadequate/undiagnostic biopsies (n) 22G/25G	Same lesion with both needles	Pancreatic head mass (%) 22G/25G or combined	Single endosonographer
**Vilmann P et al., (2013)**	Multicenter	Prospective randomized	62/73	62/64	2.8/2.7	4/5	No	N/A	No
**Lee JK et al., (2013)**	Korea	Prospective randomized	94/94	58.5/61.3	2.8/3.1	N/A	No	31/53	N/A
**Camellini L et al., (2011)**	Italy	Prospective randomized	43/41	N/A	3.6	N/A	No	72/80	No
**Siddiqui UD et al., (2009)**	USA	Prospective randomized	64/67	69/72	2.6	7/2	No	83	N/A
